# A lagging recovery: the delayed restoration of gut microbial diversity in *Rhinolophus sinicus* post-hibernation

**DOI:** 10.1186/s42523-026-00552-x

**Published:** 2026-03-20

**Authors:** Yuting Wang, Wenqing Ling, Linxia Ouyang, Sheng Zhang, Huiqian Zhou, Tao Luo, Shurong Liu, Jiale Tang, Zixian Zhou, Liping Tang, Zhilin Wang, Fuwen Wei, Guangping Huang

**Affiliations:** 1 Jiangxi Provincial Key Laboratory of Conservation Biology, College of Forestry, Jiangxi Agricultural University, Nanchang, 330045 China; 2 CAS Key Laboratory of Animal Ecology and Conservation Biology, Institute of Zoology, Chinese Academy of Sciences, Beijing, 100101 China

**Keywords:** Hibernation, Gut microbiota, Adaptation, Bat, 16S rRNA gene sequencing

## Abstract

**Background:**

Hibernation enables animals to survive extreme environments, yet gut microbiome dynamics across the full hibernation cycle remain poorly understood, particularly in chiropterans with unique physiological traits. This study aimed to precisely characterize seasonal microbial succession in wild *Rhinolophus sinicus* using 16S rRNA gene sequencing across 6 physiological stages, with a focus on taxonomic and functional shifts linked to hibernation-associated fasting and post-hibernation activity.

**Results:**

Alpha diversity followed a pronounced V-shaped trajectory, declining during hibernation and recovering only gradually—remaining suppressed in the early active stage and rebounding markedly by mid–late active stages. Beta diversity revealed a clear separation between hibernation and active phases, with physiological stage explaining 34.9% of community variation. At the phylum level, *Pseudomonadota* was the dominant taxon during hibernation, while *Bacillota* became the most abundant phylum in the active period. At the genus level, *Yokenella* was the core genus in the hibernation stage, and *Lactococcus* was the dominant genus in the active period. Functional predictions showed enrichment of lipid and amino acid metabolism during hibernation, supporting energy maintenance under fasting, while active-phase microbiota were oriented toward carbohydrate metabolism, matching increased energy demands.

**Conclusions:**

Our findings demonstrate that hibernation drives directional restructuring of the gut microbiota in *R. sinicus*, offering new insights into microecological strategies underlying bat survival under extreme conditions.

**Supplementary Information:**

The online version contains supplementary material available at 10.1186/s42523-026-00552-x.

## Background

Understanding seasonal gut microbiota dynamics is essential for elucidating host metabolic regulation [1]. The gut microbiota mediates key physiological processes, including nutrient digestion and energy acquisition, to maintain metabolic homeostasis [2, 3]. Assessing microbial composition and function in wild animals is now technically feasible and cost-effective, offering valuable insights for species conservation [4]. Seasonal variation is a major driver reshaping gut microbial structure and function [5–7], acting as a composite environmental disturbance that influences microbial remodeling [8]. Elucidating host–microbiota interactions under seasonal dynamics is therefore critical for understanding co-evolution and informing wildlife conservation strategies [9].

Hibernators display extreme seasonal adaptations and distinct metabolic phenotypes [10, 11]. Although hibernation strategies vary across mammalian taxa, metabolic and thermoregulatory features show notable convergence, and their convergent genomic changes support prolonged fasting and other hibernation demands [12]. Current research indicates that small hibernators—for example, the thirteen-lined ground squirrel—enhance microbial urease gene abundance and intestinal urea transporters during hibernation, enabling nitrogen recycling to protect muscle mass [13]. In large hibernators such as wild brown bears, gut microbial composition and metabolic potential differ markedly between active and hibernation states (with decreased *Bacillota* and increased *Bacteroidota* during hibernation), and transplantation of active-season bear microbiota improves fat accumulation and glucose tolerance in germ-free mice [14]. However, existing work has primarily focused on captive animals, leaving the seasonal microbial dynamics of wild populations insufficiently explored.

Chiropterans, distinguished by true powered flight, are widely used as model organisms for studying longevity, viral tolerance, and echolocation [15, 16]. Their hair microbiota exhibits population-level ecological and evolutionary patterns, whereas gut microbiota is shaped predominantly by individual identity and sex [17]. Most hibernating bats roost in groups during winter, maintain body temperatures only slightly above ambient levels, and exhibit periodic interbout arousals [18]. Several studies have reported seasonal shifts in gut microbial composition, diversity, and functional profiles in hibernating bat species [18–20]. Nevertheless, critical knowledge gaps remain: the year-round microbial dynamics of wild bat populations, the differentiation of gut communities across intra-hibernation stages, and the extent to which these changes couple with fluctuations in host metabolism and body temperature remain poorly understood. Hibernating bats offer a natural system for revealing host–microbiota co-evolution, yet comprehensive analyses of microbial trajectories, remodeling patterns, and environmental or host-driven influences across a full annual cycle are still lacking.

The Chinese horseshoe bat (*Rhinolophus sinicus*), distributed across southeastern and southern China and parts of Vietnam, is insectivorous, highly seasonal, and exhibits hibernation, with well-characterized genomic resources [21–23]. This makes it an ideal natural model for studying hibernation in Chiroptera. By analyzing year-round wild samples with 16S rRNA gene sequencing and functional predictions, we reveal how hibernation drives multidimensional remodeling of gut microbial structure, diversity, and metabolic capacity. These findings advance understanding of microbial ecological adaptations that support hibernation and provide a framework for elucidating microbiota-mediated mechanisms of extreme environmental adaptation in small mammals.

## Methods

### Animal capture and sample collection

From November 2024 to September 2025, wild adult male *R. sinicus* were captured from the same natural cave in Meiling Mountain, Nanchang City, Jiangxi Province, China, to minimize habitat-related confounding effects. Sex was determined based on secondary sexual characteristics. Sampling covered the complete hibernation–active cycle, with six time points in total. Individuals were selected based on body surface temperature (measured using a non-contact infrared thermometer with ± 0.1 °C accuracy) and behavioral characteristics.

The hibernation period included three experimental groups. All bats were captured using a non-disturbance approach, during which they were observed in a hanging resting posture, displaying only abdominal respiration without any signs of arousal. The groups were: the hibernation onset (OH, November, *n* = 6; body surface temperature 14.0–16.0 °C), the deep hibernation (DH, January, *n* = 4; 9.5–11.5 °C), and the late hibernation (LH, February, *n* = 4; 11.5–15.0 °C). The active period also included three groups, with bats captured in flight: the early active (EA, May, *n* = 6; 24.5–28.5 °C), the mid active (MA, July, *n* = 6; 25.5–29.0 °C), and the late active (LA, September, *n* = 6; 32.5–37.0 °C).

All experimental individuals were humanely euthanized by rapid cervical dislocation. Following euthanasia, individuals were aseptically dissected, and rectal contents were collected from the distal intestine using sterile instruments. The collected material was immediately transferred into sterile cryogenic tubes and flash-frozen in liquid nitrogen. Samples were transported on dry ice to the laboratory and stored at − 90 °C until further processing.

### DNA extraction and polymerase chain reaction (PCR) amplification

Genomic DNA was extracted from 32 rectal content samples using the DNeasy^®^ PowerSoil^®^ Pro Kit (Qiagen, Germany) following the manufacturer’s instructions. All samples were processed in a single extraction batch to minimize potential batch effects. DNA extraction was conducted in a clean laboratory environment using sterile consumables and aerosol-resistant filter tips. Negative controls were included to monitor potential contamination, including an extraction blank (no biological material) processed alongside the samples and no-template controls included in PCR. No amplification was observed in the negative controls.

The V3–V4 region of the bacterial 16S rRNA gene was amplified using primers 338 F (5′–ACTCCTACGGGAGGCAGCAG–3′) and 806R (5′–GGACTACHVGGGTWTCTAAT–3′). PCR reactions (50 µL) consisted of 25 µL Phusion^®^ High-Fidelity PCR Master Mix (New England Biolabs), 4 µL DNA template, 2 µL of each primer (10 µM), and 19 µL nuclease-free water. Thermal cycling conditions were: 95 °C for 5 min; 30 cycles of 95 °C for 30 s, 55 °C for 30 s, and 72 °C for 30 s; followed by 72 °C for 5 min. All PCR products were verified by agarose gel electrophoresis, purified, pooled in equimolar concentrations, and sequenced together on an Illumina NovaSeq 6000 platform (2 × 300 bp paired-end reads) by Shanghai Majorbio Bio-pharmTechnology Co., Ltd.

### Bioinformatics and statistical analysis

Raw sequencing data were processed using QIIME2 (v2024.2) [24] and R (v4.4.1). Paired-end reads targeting the 16S rRNA gene V3–V4 region were denoised using the DADA2 plugin with the following parameters: --p-trim-left-f 20, --p-trim-left-r 20, --p-trunc-len-f 260, and --p-trunc-len-r 240. Quality filtering was performed using DADA2 default settings, and chimeric sequences were removed using the consensus method. Singleton amplicon sequence variants (ASVs) were retained following DADA2 denoising. No additional contaminant detection tools were applied during bioinformatic processing. ASV abundance tables were exported using the feature-table plugin. Taxonomic classification was performed using the feature-classifier plugin [25] against the SILVA 138.2 database [26].

Rarefaction curves were generated using the “GUniFrac” R package [27] to assess sequencing depth. Alpha-diversity metrics (Shannon index for evenness; Chao1 for richness) were calculated and visualized using the “vegan” R package [28]. Beta-diversity was assessed using Bray–Curtis dissimilarities calculated from the ASV table (relative abundance) and visualized by principal coordinate analysis (PCoA). Homogeneity of multivariate dispersion was evaluated using betadisper (vegan) and tested with permutest (999 permutations) prior to permutational multivariate analysis of variance (PERMANOVA). Group differences in community composition were tested using PERMANOVA (adonis2 in vegan, 999 permutations). Where post hoc pairwise PERMANOVA comparisons were conducted, permutation *p*-values were adjusted for multiple testing using the Benjamini–Hochberg false discovery rate (BH-FDR) procedure, and adjusted *p*-values (*q*-values) with *q* < 0.05 were considered statistically significant. Venn diagrams were generated to visualize ASV distribution and overlap across groups, and bar plots were used to illustrate the relative abundance of the top 10 taxa at phylum and genus levels. Differences in dominant phylum-level relative abundances between hibernation and active phases were assessed using two-sided Wilcoxon rank-sum tests with BH-FDR correction. 

Differentially abundant taxa were identified using the LEfSe (Linear discriminant analysis effect size) framework implemented in the microbiomeMarker R package [29]. To reduce sparsity-driven artifacts, taxa were retained only if they were detected (non-zero) in ≥ 10% of samples and had a total count ≥ 10 across all samples. The filtered ASV table was normalized to counts per million (CPM) to account for differences in sequencing depth [29, 30]. LEfSe was then performed using the Kruskal–Wallis test to detect taxa differing among groups (*p* < 0.05), followed by pairwise Wilcoxon rank-sum tests (*p* < 0.05). Biologically discriminative features were identified based on an LDA score threshold of 2.5 [29]. Additionally, to address the compositionality of microbiome relative-abundance data, the filtered ASV table was converted to within-sample relative abundances (sum-to-one) [31]. A pseudocount (1 × 10⁻⁶) was added to handle zero values, and data were then centered log-ratio (CLR) transformed. Differences between the hibernation and active groups were assessed using two-sided Wilcoxon rank-sum tests on CLR-transformed values, with BH-FDR correction (*q* < 0.05).

KEGG functional profiles predicted by PICRUSt2 (v2.6.2) [32] were treated as compositional data. KO-level abundances were aggregated to KEGG pathway levels (L3/L2/L1), and when a KO mapped to multiple pathways, its abundance was evenly distributed across mapped pathways. Within each sample, pathway abundances were converted to relative abundances (closure to sum to 1) and transformed using CLR with a pseudocount of 1 × 10⁻⁶. Differential abundance between hibernation and active groups was assessed using Wilcoxon rank-sum tests on CLR-transformed values. Multiple testing correction was performed using the Benjamini–Hochberg false discovery rate method, and adjusted *p*-values (*q*-values) were reported. Pathways with *q* < 0.05 were considered statistically significant. For interpretability, mean ± SD (%) and log₂ fold change (hiber/activate) were calculated from relative abundances.

## Results

### Gut microbial charactering of the Chinese horseshoe bat

**Fig. 1:**
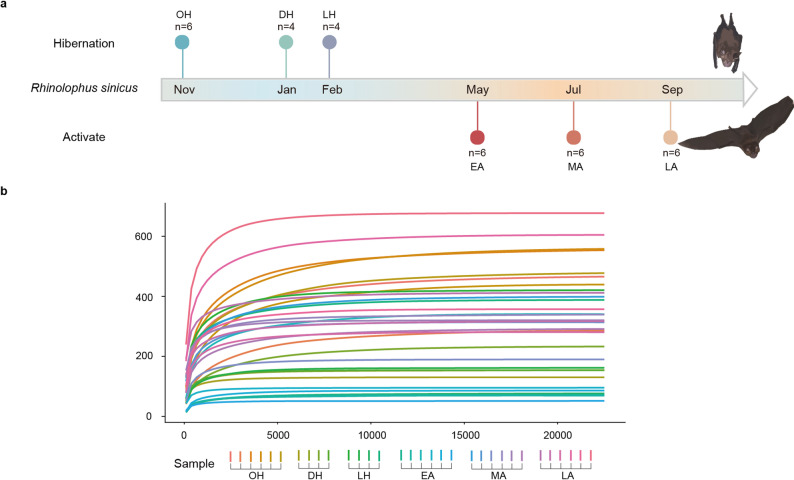
Seasonal sampling design and rarefaction curves of gut microbiota in *R. sinicus*. (**a**) Schematic diagram of the seasonal sampling design for *R. sinicus* throughout the annual cycle, including three hibernation stages (OH: hibernation onset, DH: deep hibernation, LH: late hibernation) and three active stages (EA: early active, MA: mid active, LA: late active), with sample sizes indicated for each group. (**b**) Rarefaction curves based on ASVs of gut microbiota across all sampling groups. The curves approach saturation, indicating that the sequencing depth is sufficient to reflect the true community structure of the gut microbiota

During hibernation, *R. sinicus* exhibited marked physiological changes, including significantly reduced metabolic rate and activity, accompanied by pronounced body temperature fluctuations (Fig. 1a). Sequencing the V3–V4 region of the 16S rRNA gene from 32 adult male bats yielded 1,618,110 high-quality sequences (average 50,566 reads per sample), clustered into 7,647 ASVs. Rarefaction curve analysis indicated that sequencing depth was sufficient to capture the full diversity of gut microbial communities (Fig. 1b). Taxonomic annotation revealed sequences assigned to 43 phyla, 88 classes, 192 orders, 297 families, and 750 genera, highlighting the high genus-level diversity that supports metabolic functions during hibernation.

**Fig. 2:**
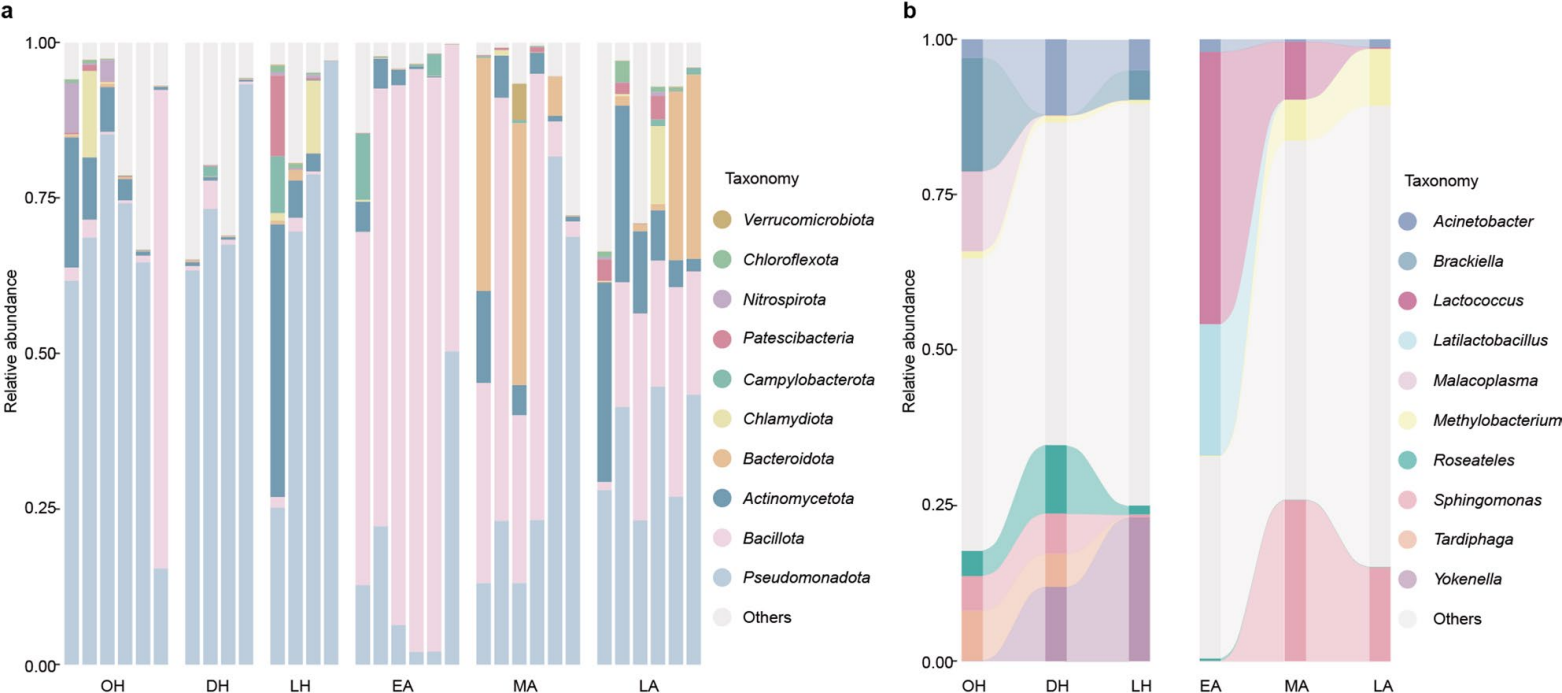
Seasonal dynamics of gut microbial composition in *R. sinicus* at phylum and genus levels. (**a**) Relative abundance of the top 10 bacterial phyla across different seasonal groups (OH: hibernation onset, DH: deep hibernation, LH: late hibernation, EA: early active, MA: mid active, LA: late active). (**b**) Relative abundance of the top 10 bacterial genera across different seasonal groups. These panels illustrate the distinct seasonal shifts in gut microbial composition at both phylum and genus levels, reflecting the adaptive restructuring of the microbiota to the host’s physiological changes

At the phylum level, the gut microbiota was dominated by *Pseudomonadota*, *Bacillota*, and *Actinomycetota* (Fig. 2a), reflecting conserved patterns observed in *Rhinolophus* species and indicating baseline community stability [20]. Two-sided Wilcoxon rank-sum tests with BH-FDR correction showed significant seasonal differences for the two major phyla: *Pseudomonadota* was higher during hibernation (67.01 ± 22.55%) than during the active period (29.27 ± 22.10%; *q* = 0.0004), whereas *Bacillota* increased from 6.77 ± 20.23% in hibernation to 43.59 ± 30.93% in the active period (*q* = 0.0001).

At the genus level, the most abundant taxa across all samples included *Lactococcus* (10.01 ± 22.17%), *Sphingomonas* (9.63 ± 16.42%), *Yokenella* (4.41 ± 18.17%), *Brackiella* (4.02 ± 13.86%), and *Latilactobacillus* (3.95 ± 16.02%) (Fig. 2b). Seasonal shifts in dominant genera were pronounced. During hibernation, the onset stage (OH) was dominated by *Brackiella* (18.28 ± 28.91%), *Malacoplasma* (12.83 ± 31.35%), and *Tardiphaga* (8.02 ± 6.30%); the deep hibernation (DH) was dominated by *Acinetobacter* (12.20 ± 7.56%), *Yokenella* (11.99 ± 23.79%) and *Roseateles* (11.04 ± 6.34%); and the late hibernation (LH) was dominated by *Yokenella* (23.20 ± 46.31%). During the active period, the early active (EA) was dominated by *Lactococcus* (43.80 ± 32.73%, capable of degrading insect chitin [33]) and *Latilactobacillus* (21.07 ± 34.03%, short-chain fatty acid producers [34, 35]), whereas the mid active (MA) and the late active (LA) were dominated by *Sphingomonas* (25.93 ± 29.62% and 15.10 ± 14.33%, respectively), with the LA also enriched in *Methylobacterium* (9.17 ± 6.43%), reflecting adaptation to diverse dietary resources.

### Variations in gut microbiota diversity and community across hibernation cycle

**Fig. 3:**
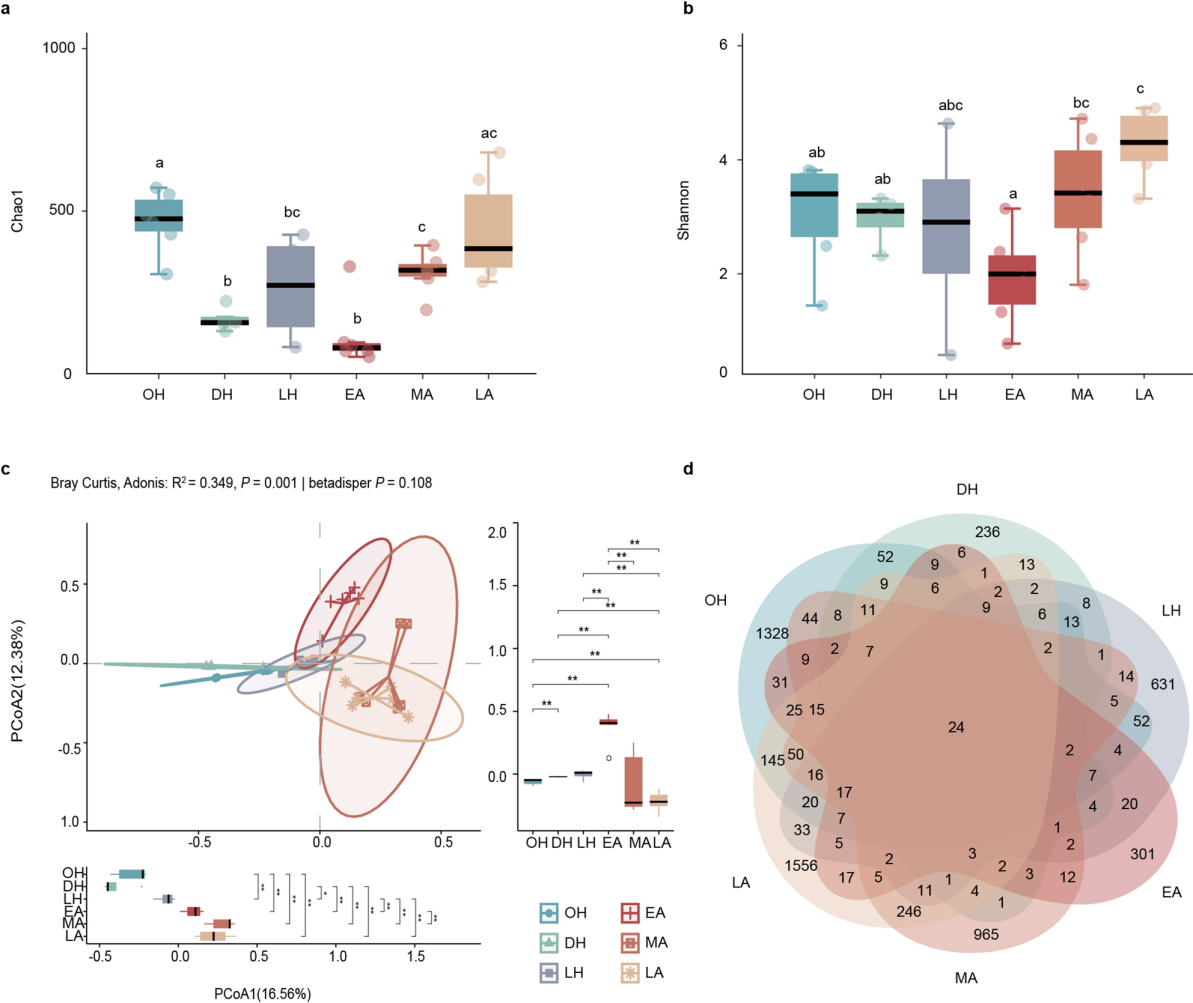
Seasonal variations in gut microbial diversity and community structure of *R. sinicus*. (**a**) Box plot of Chao1 index (reflecting species richness) across different seasonal groups. (**b**) Box plot of Shannon index (reflecting community evenness) across different seasonal groups. Different lowercase letters indicate significant differences (*p* < 0.05). (**c**) PCoA based on Bray-Curtis distance, with the inset showing the results of PERMANOVA (Adonis, R² = 0.349, *P* = 0.001) and pairwise comparisons. (**d**) Venn diagram illustrating the number of shared and unique ASVs among different seasonal groups.

To elucidate the remodeling effects of hibernation on the gut microbiota, we analyzed community richness, diversity, and composition across the annual cycle. Microbial diversity fluctuated significantly throughout the year. Chao1 richness ranged from 51 to 679.96, and Shannon diversity from 0.57 to 4.91, with the late active (LA) group reaching the annual peak. The mid active (MA) and the hibernation onset (OH) also showed high species richness and diversity (Fig. 3a and b). Overall, microbial diversity declined throughout hibernation, reaching a nadir, and gradually recovered during the active period. No significant differences were observed among hibernation subgroups (OH, DH, LH), indicating that seasonal host metabolism and diet strongly regulate gut microbial dynamics (Fig. 3a and b).

PCoA based on Bray–Curtis dissimilarities showed separation among groups (Fig. 3c), with the first two axes explaining 16.56% (PCoA1) and 12.38% (PCoA2) of the variation. PERMANOVA indicated a significant effect of grouping on community structure (R² = 0.349, *P* = 0.001; 999 permutations). Homogeneity of multivariate dispersion was supported (betadisper, *P* = 0.108; 999 permutations), and pairwise dispersion tests were not significant after BH-FDR correction (all *q* > 0.05; Supplementary Table 1), indicating comparable within-group variability. Pairwise post hoc PERMANOVA comparisons with BH-FDR correction showed that each hibernation subgroup differed significantly from each active subgroup (all *q* ≤ 0.015; Supplementary Table 2). Venn diagram analysis demonstrated substantial seasonal heterogeneity: the LA group harbored 1,556 unique ASVs, OH 1,328, DH 236, with 24 ASVs shared across all six groups (Fig. 3d), reflecting both stage-specific microbiota and conserved core functions.

### Seasonal differentiation of gut microbial taxa and functional pathways

**Fig. 4:**
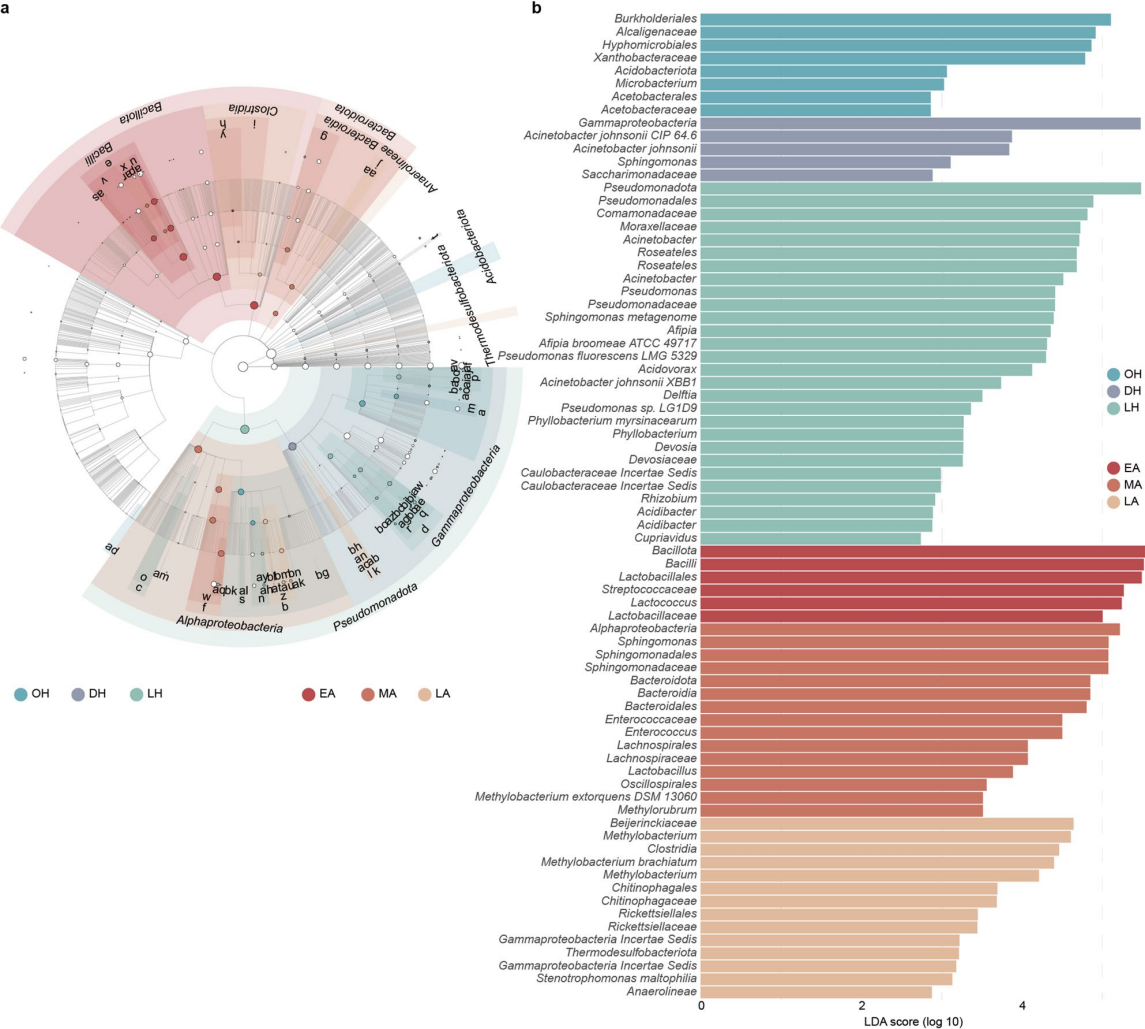
Identification of differential gut microbial taxa across seasonal groups in *R. sinicus* by LEfSe analysis. (**a**) Taxonomic cladogram showing the differentially abundant microbial taxa (from phylum to species level) among different seasonal groups. Nodes with different colors represent taxa enriched in the corresponding group, and the size of the node is proportional to its relative abundance. (**b**) LDA score histogram of differentially abundant taxa, with an LDA score threshold of 2.5. Taxa with higher LDA scores have greater discriminative power between groups

LEfSe analysis identified pronounced differentiation in gut microbial taxa across physiological stages, with 78 taxa showing significant differences from phylum to genus level (Fig. 4a and b, Supplementary Table 3). Each stage exhibited distinct enriched taxa, and no taxa were consistently enriched across all stages. During hibernation, *Pseudomonadota* was the predominant. The hibernation onset group (OH) was enriched in *Burkholderiales* (LDA > 4.8), whereas the deep hibernation group (DH) was enriched in *Pseudomonas* and *Acinetobacter*. In the active period, the relative abundance of *Bacillota* and *Bacteroidota* increased. The early active group (EA) was enriched in *Bacillot*a, the mid active group (MA) was enriched in *Sphingomonadales*, and the late active group (LA) was enriched in *Chitinophagaceae* and *Methylobacterium* (Fig. 4a and b).

Genus-level aggregation of CLR-transformed differential abundance results (two-sided Wilcoxon rank-sum tests with BH-FDR correction, *q* < 0.05) indicated that the active group was enriched in *Adlercreutzia*, whereas the hibernation group was enriched in several *Pseudomonadota*-associated genera, including *Acinetobacter*, *Pseudomonas*, *Acidovorax*, and *Sphingomonas* (Supplementary Table 4).

**Fig. 5:**
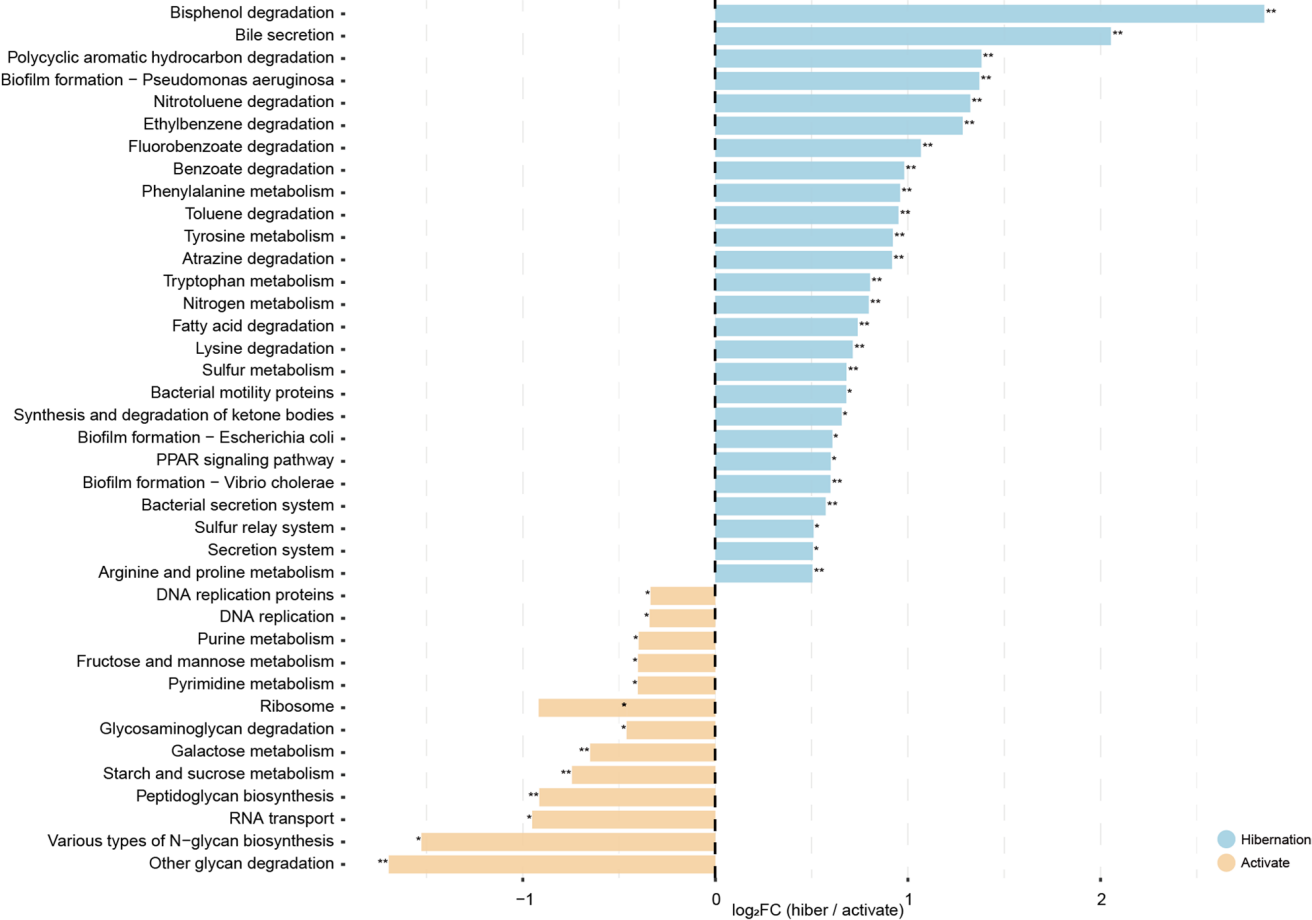
Differential functional pathways of gut microbiota between hibernation and active periods based on PICRUSt2 prediction. Significantly differential functional pathways of the gut microbiota between the hibernation and active periods in *R. sinicus* (analyzed by logarithmic fold change, log₂(FC)). Pathways enriched in the hibernation period are shown in blue, while those enriched in the active period are in orange. Asterisks indicate statistical significance (* represents *q* < 0.05, ** represents *q* < 0.01)

PICRUSt2-based functional prediction annotated 9,269 KEGG pathway-associated genes (Fig. 5). Differential analysis of CLR-transformed pathway profiles showed significant differences at KEGG level 1 (2 categories, *q* < 0.05) and level 2 (14 categories, *q* < 0.05), including 4 categories with *q* < 0.01. Pathways related to lipid metabolism and amino acid metabolism were enriched during hibernation, whereas carbohydrate metabolism pathways were enriched during the active period (Fig. 5).

## Discussion

The gut microbiota acts as a pivotal functional interface between the host and the external environment, dynamically adjusting its composition and functional potential in response to host physiology and environmental pressures [36]. Such adaptive modulation is essential for hibernators to withstand prolonged fasting, low temperatures, and metabolic suppression [14]. By integrating 16S rRNA gene sequencing and functional predictions, our study systematically reveals hibernation-driven remodeling of the gut microbiota in *R. sinicus* across structural, diversity, and functional dimensions. These findings advance our understanding of host–microbiota co-adaptation in hibernating mammals and provide empirical evidence for the evolutionary conservation and ecological plasticity of gut microbiota within the genus *Rhinolophus*.

Microbial richness, diversity, and community structure are key indicators of host–microbiota responses [37]. Unlike prior studies focusing on peak-diversity stages [19, 38, 39], our full-cycle monitoring reveals a lagged recovery of alpha diversity during the early active (EA) stage, which peaks only in the late active (LA) stage. Diversity declines throughout hibernation, with no significant differences among subgroups (OH, DH, LH), and rises markedly only in mid–late activity, consistent with patterns observed in other hibernators, such as Arctic ground squirrels [19, 38, 39]. Beta-diversity indicated that hibernation stabilizes gut communities, whereas the active period promotes diversification through dietary complexity and metabolic reactivation. We hypothesize that this lag reflects host resource allocation, prioritizing tissue repair and immune recovery over immediate microbial repopulation. Overall, these findings highlight hibernation’s central role in shaping gut microbial ecology. Integrating multi-omics approaches—including metagenomics and transcriptomics—will be crucial to elucidate the molecular mechanisms underlying host–microbiota interactions across hibernation–activity transitions.

The composition and function of the gut microbiota are tightly linked to host health, and the extreme environmental stress imposed by hibernation drives microbial communities toward specialization on endogenous nutrients [12]. In *R. sinicus*, the gut microbiota functions as a key mediator of host adaptation to seasonal physiological changes, coordinating shifts across phylum, genus, and functional pathway levels. Our data revealed pronounced taxonomic specialization during hibernation, with the relative abundance of *Pseudomonadota*—a resilient core phylum, showing a significant increase during the hibernation phase. Upon return to the active period, concurrent with the availability of insect prey (e.g., Coleoptera and Lepidoptera) and elevated host metabolic rate, the gut microbial community rapidly shifted toward the utilization of exogenous substrates, resulting in *Bacillota* becoming the dominant phylum. This seasonal turnover of dominant phyla exemplifies a common adaptive strategy among hibernators, as documented in brown bears [14] and Arctic ground squirrels [39], reflecting convergent functional specialization under endogenous nutrient limitation. Specifically, the high abundance of *Pseudomonadota* during hibernation is an adaptive response to the host’s low body temperature and reduced metabolic rate [40], while the enrichment of *Bacillota* in the active period enhances fat absorption to satisfy the high energy requirements of sustained flight [41], a unique physiological trait of Chiropterans. Notably, within the genus *Rhinolophus*, *R. ferrumequinum* also exhibits increased *Pseudomonadota* and decreased *Bacillota* during hibernation [19], further highlighting the evolutionary conservation of gut microecological adaptation in this clade and its optimized role in maintaining intestinal homeostasis and energy balance.

The genetic diversity of the gut microbiota shapes community structure and provides enzymatic and metabolic capacities absent from the host genome, supporting adaptation to complex environments [36]. In the active period of *R. sinicus*, genera such as *Lactococcus* and *Latilactobacillus* dominate, efficiently degrading exogenous substrates like insect chitin and polysaccharides. Their activities, including short-chain fatty acid production and enhanced lipid absorption [42], supply energy for high-demand processes such as sustained flight and reproduction [34, 35]. During hibernation, *Roseateles* is enriched; some strains produce peptide antibiotics or harbor biosynthetic gene clusters, potentially suppressing harmful bacteria and maintaining gut homeostasis [43]. *Pseudomonas*, the key enriched genus in deep hibernation, exhibits cold tolerance and the ability to utilize endogenous substrates [44], which further adapts to the host’s fasting and low-temperature state during hibernation. Opportunistic pathogens, including *Serratia* and *Citrobacter*, are also present, likely reflecting environmental exposure through prey or water contact [20]. Many strains remain unclassified at the genus level, and functional validation in pure culture is lacking. Future studies integrating metagenomics and culturomics are needed to clarify the roles of key microbes and their contributions to host health, providing deeper insight into host–microbiota interactions.

Gut microbial communities are assembled under defined ecological selection, and functionally specialized taxa contribute to host seasonal physiology by executing specific metabolic programs [45]. Integrating microbial composition with PICRUSt2 predictions, we observed that hibernation enriches resilient taxa and functional pathways including lipid metabolism, amino acid metabolism, and bacterial secretion systems, aligning with the host’s reliance on fat mobilization and protein conservation under low-temperature, nutrient-limited conditions. During the active period, carbohydrate metabolism pathways (e.g., galactose, starch, and sucrose metabolism) and one-carbon metabolism are preferentially activated, facilitating rapid degradation of chitin and glycogen to release glucose and providing essential intermediates for nucleic acid synthesis and protein metabolism. This functional seasonality supports both microbial proliferation and host physiological demands under high metabolic activity. Conserved microbe–host metabolic regulatory pathways have been reported in other hibernators, such as thirteen-lined ground squirrels, where urease gene-enriched gut microbiota enhance urease activity and nitrogen recycling during hibernation [13]. While our study establishes strong correlations between microbial function and host physiology, causal relationships remain to be fully elucidated. Future integration of metabolomics to monitor key metabolites (e.g., fatty acids) and microbial transplantation experiments will be critical to directly test whether microbiota-mediated functional changes modulate host energy metabolism, thereby refining our understanding of the hibernation–host–microbiota interaction network.

This study is based on 16S rRNA gene amplicon sequencing; therefore, functional profiles inferred using PICRUSt2 should be interpreted as predictions rather than direct measurements of genomic content or metabolic activity. Reference databases may have limited coverage of bat-associated microbial lineages, which can reduce the accuracy of pathway inference and may fail to capture host-specific functional potential. In addition, bat gut samples can contain low and highly variable fractions of microbial DNA [46], potentially affecting sequencing depth, taxonomic resolution (particularly at finer ranks), and downstream functional inference. Accordingly, the functional results reported here are best considered hypothesis-generating, and future work integrating shotgun metagenomics, meta-transcriptomics, metabolomics, and standardized positive/negative control validation will be important to validate key functional shifts and resolve strain-level functional capacities.

## Conclusions

In summary, this study demonstrates the characteristic V-shaped dynamic pattern of the gut microbiota in *R. sinicus*, indicating that the restoration of host physiological status is a prerequisite for gut microbial reconstitution. This research not only unveils fundamental principles governing host-microbe interactions but also furnishes valuable insights into mammalian adaptation to extreme environments.

## Supplementary Information

Below is the link to the electronic supplementary material.

Supplementary Material 1: Supplementary Table 1. Pairwise tests of multivariate dispersion (betadisper) based on Bray–Curtis distances: permutation *p*-values and BH-adjusted *q*-values. Supplementary Table 2. Pairwise PERMANOVA (Bray–Curtis) results among groups with BH-FDR–adjusted *q*-values. Supplementary Table 3. Explanation of character in LEfSe analysis. Supplementary Table 4. Genus-level CLR-based differential abundance (hiber vs ac-tive; Wilcoxon + BH-FDR, *q* ≤ 0.05).

## Data Availability

The raw sequencing data (FASTQ files) generated in this study have been deposited in the Genome Sequence Archive (GSA) at the China National Center for Bio-information (CNCB-NGDC) under BioProject accession number PRJCA053117, with the primary accession number CRA034954.
